# A novel dual‐marker expression panel for easy and accurate risk stratification of patients with gastric cancer

**DOI:** 10.1002/cam4.1522

**Published:** 2018-05-07

**Authors:** Mitsuro Kanda, Kenta Murotani, Haruyoshi Tanaka, Takashi Miwa, Shinichi Umeda, Chie Tanaka, Daisuke Kobayashi, Masamichi Hayashi, Norifumi Hattori, Masaya Suenaga, Suguru Yamada, Goro Nakayama, Michitaka Fujiwara, Yasuhiro Kodera

**Affiliations:** ^1^ Department of Gastroenterological Surgery (Surgery II) Nagoya University Graduate School of Medicine Nagoya Japan; ^2^ Clinical Research Centre Aichi Medical University Hospital Nagakute Japan

**Keywords:** Biomarker, expression panel, gastric cancer, prognosis, recurrence

## Abstract

Development of specific biomarkers is necessary for individualized management of patients with gastric cancer. The aim of this study was to design a simple expression panel comprising novel molecular markers for precise risk stratification. Patients (*n* = 200) who underwent gastrectomy for gastric cancer were randomly assigned into learning and validation sets. Tissue mRNA expression levels of 15 candidate molecular markers were determined using quantitative PCR analysis. A dual‐marker expression panel was created according to concordance index (C‐index) values of overall survival for all 105 combinations of two markers in the learning set. The reproducibility and clinical significance of the dual‐marker expression panel were evaluated in the validation set. The patient characteristics of the learning and validation sets were well balanced. The C‐index values of combinations were significantly higher compared with those of single markers. The panel with the highest C‐index (0.718) of the learning set comprised *SYT8* and *MAGED2*, which clearly stratified patients into low‐, intermediate‐, and high‐risk groups. The reproducibility of the panel was demonstrated in the validation set. High expression scores were significantly associated with larger tumor size, vascular invasion, lymph node metastasis, peritoneal metastasis, and advanced disease. The dual‐marker expression panel provides a simple tool that clearly stratifies patients with gastric cancer into low‐, intermediate‐, and high risk after gastrectomy.

**Surgical Relevance**

**What is already known**

Commercially available multigene expression assays for several malignancies contribute to clinical decision‐making, but those for gastric cancer must be developed.

**What is new**

Here, we developed a novel dual‐marker expression panel that enables clinicians to stratify patients into low‐, intermediate‐, and high‐risk groups after gastrectomy for gastric cancer. Moreover, the expression panel demonstrated superior predictive performance compared with single component and conventional tumor markers (carcinoembryonic antigen and carbohydrate antigen 19‐9).

**Potential impact on future practice**

Excessive postoperative intervention of monitoring and treatment can be avoided for patients at low risk, leading to reduced patient burden and medical costs. Alternatively, intensive systemic surveillance and aggressive perioperative therapy could be considered for patients at high risk, anticipating early recurrence and an adverse prognosis. Our study concept utilized knowledge obtained from identification of single molecular markers to create a dual‐marker expression panel, which likely will contribute to precision medicine designed to manage gastric cancer.

## Introduction

Gastric cancer is one of the deadliest tumors worldwide 1. Even with the decline in incidence, the mortality rate remains high, which mainly explained the extreme heterogeneity of this disease that varies widely in its molecular and clinical characteristics 1, 2, 3. Given the very high clinical burden of gastric cancer worldwide, development of informative biomarkers is mandatory to achieve early diagnosis, accurate prediction of prognosis, disease monitoring, and evaluation of treatment responses. Despite the availability of numerous molecular markers that are differentially expressed in patients with gastric cancer, most investigators focused only on individual markers with limited performance for predicting differences in the biology of individual tumors 4, 5, 6. Ultimately, the availability of multiple markers likely will contribute to the efficacy of precision medicine.

The concept of combining multiple markers is considered the best alternative for overcoming the limitations of single markers and will maximize their clinical usefulness 7, 8. For example, the predictive value of disease recurrences achieved using the Oncotype DX Breast Cancer Assay (Genomic Health), a multigene panel comprising 21 genes, was demonstrated by a large clinical trial 9. To our knowledge, a multigene assay kit for gastric cancer has not been similarly validated. Lee et al. 10 developed a recurrence risk score assay comprising six molecular markers and reported its high predictive performance as being an independent prognostic factor.

Understanding the biological characteristics associated with the inherent heterogeneity of gastric cancer using panels integrating multiple molecular markers may reflect individual cancer phenotypes and significantly improve patient care. In this context, we also reported a risk model consisted of the four molecular markers to prognosticate patients with gastric cancer previously 11. Although inclusion of multiple factors in the expression panels may enhance the predictive performance, the clinical utility of multigene panels is limited by increasing a burden of effort, cost, and the complexity in the process of statistics and scoring. Given that, it is worth challenging to develop a dual‐marker expression panel being simple and consisted of only two markers, but having a high predictive performance. Since 2014, researchers at Nagoya University discovered 15 prognostic biomarkers for gastric cancer 12, 13, 14, 15, 16, 17, 18, 19, 20, 21, 22, 23, 24, 25, 26. This study aimed at testing the hypothesis that a combination of molecular markers can be used to establish a dual‐marker expression panel that will improve stratification of patients with gastric cancer.

## Methods

### Patients, sample collection, and ethics

This study included 200 patients who underwent gastrectomy for gastric cancer at Nagoya University Hospital between November 2001 and December 2014. Primary gastric cancer tissues and corresponding adjacent noncancerous gastric tissues were obtained from resected specimens. Tissue samples were immediately frozen in liquid nitrogen and stored at −80°C until use for RNA extraction. Approximately 5 mm^2^ was extracted from each tumor sample, avoiding necrotic tissue by gross observation, and only samples confirmed to comprise more than 80% tumor components by H&E staining were included in this study. Corresponding normal adjacent gastric mucosa samples were obtained from the same patient and were collected >5 cm from the tumor edge. This study conformed to the ethical guidelines of the Declaration of Helsinki and was approved by the Institutional Review Board of Nagoya University, Japan (approval number 2014‐0043). Written informed consent for use of clinical samples and data, as required by the institutional review board, was obtained from all patients.

### Measurement of mRNA expression levels of molecular markers

RNA was extracted from 200 pairs of gastric tissues using an RNeasy Mini Kit (Qiagen, Hilden, Germany), and a quality check of RNA samples was conducted before generating cDNAs. The ratios of absorbance at 260 and 280 nm of the RNAs ranged from 1.8 to 2.0. Total RNA (10 *μ*g per sample) was isolated and used as template for cDNA synthesis. Quantitative real‐time RT‐PCR (qRT‐PCR) was performed to determine mRNA expression levels using an ABI StepOnePlus Real‐Time PCR System (Applied Biosystems, Foster City, CA). Technical replicates were performed in triplicate for all samples. Fifteen candidate molecular markers of gastric cancer (Table 1) were subjected to mRNA expression analysis. The level of glyceraldehyde‐3‐phosphate dehydrogenase (*GAPDH*) mRNA was quantified in each sample and used to normalize the data. Primer sequences used in this study are listed in Table S1. Patients were categorized into two groups using the cutoff values from our previous studies (Table 1).

**Table 1:** List of candidate markers

Function	Symbol	Full name	Optimal cutoff^a^
Cell adhesion factor	*ANOS1*	Anosmin‐1	C median
	*DPYSL3*	Dihydropyrimidinase‐like 3	C median
Immunomodulatory factor	*BTG1*	BTG antiproliferation factor 1	C/N < 1/3
	*MZB1*	Marginal zone B and B1 cell‐specific protein	C median
	*SAMSN1*	SAM domain, SH3 domain, and nuclear localization signals 1	C median
Membrane trafficking protein	*DENND2D*	DENN domain containing 2D	C/N < 0.5
	*GPR155*	G protein‐coupled receptor 155	C 0.0009
	*MFSD4*	Major facilitator superfamily domain containing 4	C = 0.006
	*SYT8*	Synaptotagmin VIII	C = 0.005
Metabolic enzyme	*PDSS2*	Decaprenyl diphosphate synthase subunit 2	C/N < 0.5
Transcription factor	*FAM46C*	Family with sequence similarity 46, member C	C median
	*PRMT5*	Protein arginine methyltransferase 5	C median
Tumor‐specific antigen	*MAGED2*	MAGE family member D2	C/N > 1
	*NRAGE*	Neurotrophin receptor‐interacting melanoma antigen‐encoding protein	C mean
Unknown	*TUSC1*	Tumor suppressor candidate 1	C 1st quartile

a From references 11, 12, 13, 14, 15, 16, 17, 18, 19, 20, 21, 22, 23, 24, 25.

### Development and validation of a dual‐marker expression panel

**Figure 1:**
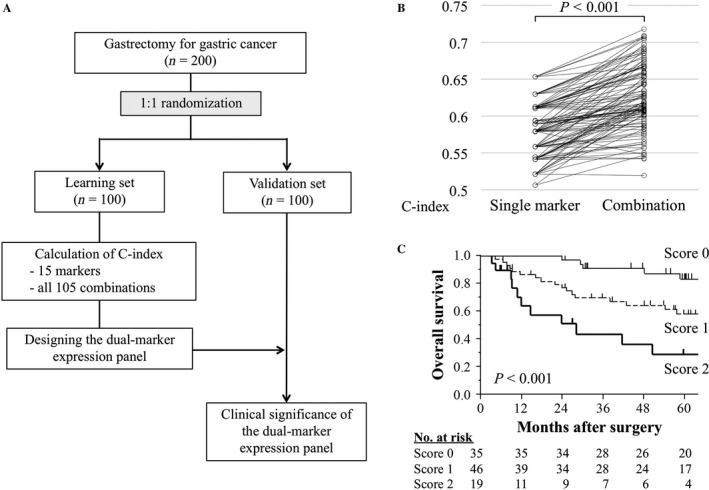
Development of a dual‐marker expression panel. (A) Study flowchart. (B) The C‐index values were significantly higher in combinations of each of two markers compared with those of single markers (*P *< 0.001). (C) Overall survival of patients in the learning set according to the expression score.

Using a table of randomly generated numbers, the 200 patients were equally divided into the learning and validation sets. To design a dual‐marker expression panel, concordance index (C‐index) values for overall survival were calculated for all 105 possible combinations of each of two markers in the learning set. Using the expression panel that yielded the highest C‐index, patients were classified as score 0 (both negative), 1 (one of two positive), or 2 (both positive). To test the reproducibility of the dual‐marker expression panel, predictive performance was evaluated in the validation set (Fig. 1A). To evaluate the predictive performance of the dual‐marker expression panel for disease recurrences after curative gastrectomy, patients with stage I‐III gastric cancer were included in the subgroup analysis (patients with stage IV gastric cancer were excluded) to analyze disease‐free survival and recurrence patterns in the validation set.

For external data validation, an integrated dataset comprising 1065 patients from three major cancer research centers (Berlin, Bethesda, and Melbourne datasets) was accessed at http://kmplot.com/analysis/ 27. We used this database to validate the predictive performance of the components of the dual‐marker expression panel.

### Statistical analysis

The Cox regression model was used to evaluate the overall survival (hazard ratio) associated with each variable. The prediction score was internally validated using the C‐index that indicates the probability of concordance between predicted and observed survival, with C = 0.5 for random predictions and C = 1 for a perfect discrimination score. The C‐index was evaluated using the learning set, bootstrapping 10,000 resamples 28. Overall and disease‐free survival rates were estimated using the Kaplan–Meier method, and the differences in survival curves were evaluated using the log‐rank test. The qualitative chi‐square and quantitative Mann–Whitney tests were used to compare two groups. Multivariable regression analysis was conducted using the Cox proportional hazards model, and variables with *P *< 0.05 were entered into the final model. Statistical analysis was performed using JMP 10 software and SAS 9.4 (SAS Institute Inc., NC). *P *< 0.05 indicates a statistically significant difference.

## Results

### Development of a dual‐marker expression panel

There were no significant differences in demographics, tumor location, macroscopic type, and disease stage between the learning and validation sets (Table S2). The C‐index values were higher in 98 (93.3%) combinations compared with the single markers (Fig. 1B). Among 105 combinations of each of two markers, the panel with the highest C‐index (0.718; 95% confidence interval 0.639–0.791) included synaptotagmin VIII (*SYT8*) and melanoma antigen gene family member D2 (*MAGED2*) (Table S3). According to our previous reports, high *MAGED2* was defined as follows: when the expression level in gastric cancer tissue was higher than that in the corresponding normal adjacent tissue 17. Patients were classified as high *SYT8* when *SYT8* mRNA expression levels (*SYT8*/*GAPDH*) in gastric cancer tissues were 0.005 or greater 24. This dual‐marker expression panel clearly stratified patients with favorable, moderate, and poor overall survival (Fig. 1C).

### Validation of the dual‐marker expression panel

**Figure 2:**
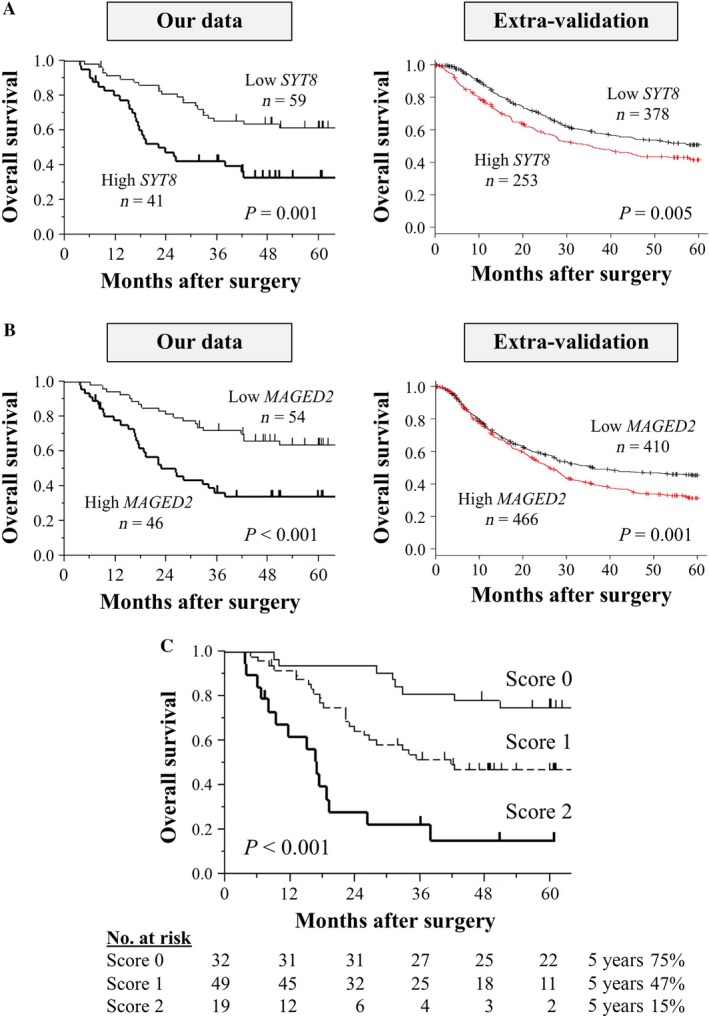
Performance of the dual‐marker expression panel in the validation set. (A) Overall survival of patients according to *SYT8* expression using our data and those of the external validation cohort. (B) Overall survival of patients according to *MAGED2* expression using our data and those of the external validation cohort. (C) Overall survival of patients in the validation set according to the expression score.

The reproducibility of the panel was evaluated in the validation set. First, the prognostic impact of *SYT8* or *MAGED2* was evaluated in the two databases described above. Patients in both cohorts with high versus low levels of *SYT8* mRNA experienced significantly shorter overall survival (Fig. 2A). Similarly, patients in both cohorts with high versus low levels of *MAGED2* mRNA were more likely to have a poorer prognosis (Fig. 2B). The prognostic values of the preoperative serum markers carcinoembryonic antigen (CEA) and carbohydrate antigen (CA) 19‐9 in the validation set are shown in Figure S1. Neither marker exhibited the equivalent stratifying performance compared with the components of the dual‐marker expression panel.

Reproducing the results of the learning set, the overall survival curves of patients with scores 0, 1, or 2 were clearly distinguished (Fig. 2C), indicating that the dual‐marker expression panel clearly stratified patients into low‐, intermediate‐, and high risk of long‐term survival after gastrectomy. When evaluating the association between the score and clinicopathological parameters, there were no significant differences associated with age, sex, or tumor differentiation. In contrast, a higher score is significantly associated with larger tumor size, vascular invasion, lymph node metastasis, peritoneal metastasis, and advanced disease stage (Table 2).

**Table 2:** Association between expression scores and clinicopathological parameters in the validation set

Variables	Score 0	Score 1	Score 2	*P*
Age				
<70 years	15	29	11	0.532
≥70 years	17	20	8	
Sex				
Male	24	35	12	0.670
Female	8	14	7	
CEA (ng/mL)				
≤5	26	36	14	0.693
>5	6	13	5	
CA19‐9 (IU/mL)				
≤37	28	36	13	0.188
>37	4	13	6	
Tumor location				
Entire	1	3	5	0.012
Upper third	9	8	2	
Middle third	13	12	2	
Lower third	9	26	10	
Tumor size (mm)				
<50	16	9	4	0.008
≥50	16	40	15	
Tumor depth (UICC)				
pT1–3	14	23	6	0.507
pT4	18	26	13	
Differentiation				
Differentiated	14	17	4	0.245
Undifferentiated	18	32	15	
Lymphatic involvement				
Absent	5	6	1	0.502
Present	27	43	18	
Vascular invasion				
Absent	16	22	2	0.007
Present	16	27	17	
Infiltrative growth type				
Invasive growth	8	20	13	0.009
Expansive growth	24	29	6	
Lymph node metastasis				
Absent	13	14	1	0.012
Present	19	35	18	
Peritoneal metastasis				
Absent	27	34	11	0.005
Present	5	15	8	
Synchronous hepatic metastasis				
Absent	31	48	16	0.107
Present	1	1	3	
UICC stage				
I	9	7	0	0.022
II	5	9	1	
III	11	16	7	
IV	7	17	11	

CEA, carcinoembryonic antigen; CA19‐9, carbohydrate antigen 19‐9; UICC, Union for International Cancer Control.

### Association between the expression score and disease recurrence after curative gastrectomy

**Figure 3:**
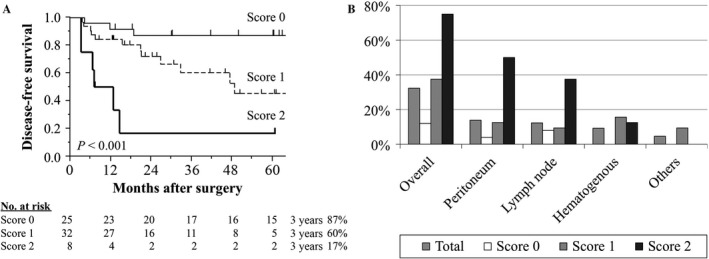
Disease recurrence and expression scores. (A) Disease‐free survival of patients with an expression score = 0, 1, or 2. (B) Distribution of recurrence patterns according to the expression score.

In patients with stage I‐III gastric cancer (*n* = 75), disease‐free survival rates gradually decreased with increasing score (Fig. 3A). Multivariable analysis revealed that expression score was an independent prognostic factor for disease‐free survival after curative gastrectomy (hazard ratio 4.24, 95% confidence interval 1.42–18.3, *P *= 0.008; Table S4). The prevalence of peritoneal and nodal recurrences increased concurrently with the expression score (Fig. 3B). Hematogenous recurrences were not observed in patients with score 0 (Fig. 3B).

## Discussion

Molecular targets for therapy are emerging rapidly, and the development of clinical tests that simultaneously screen for multiple targets is particularly important 29, 30, 31. Here, we developed a dual‐marker expression panel that stratified patients into low‐, intermediate‐, and high‐risk groups after they underwent gastrectomy for gastric cancer. The strengths of the panel are as follows: The novel panel comprised two novel molecular markers. The panel identified patients at high or low risk. The results were reproducible, as demonstrated through analyses of randomly assigned members of two cohorts as well as through an external validation cohort.

To identify a dual‐marker expression panel with the greatest predictive value, the C‐index value was calculated for each of 105 combinations of each of two molecular markers. As expected, a higher C‐index was associated with most combinations compared with that of each single marker. Among the combinations, we selected a dual‐marker expression panel comprising *SYT8* and *MAGED2*. The single use of *SYT8* and *MAGED2* exhibited superior predictive performance compared with CEA or CA19‐9, each of which is currently used as a marker of gastric cancer.

The reliability of these data was documented using the extra validation cohort, although the survival differences were more apparent in our data because of a large proportion of stage IV patients (36% and 35% of patients were diagnosed as stage IV gastric cancer in the learning and validation sets, respectively) 27. However, precise patient stratification is difficult using two groups distributed below or above the cutoff, respectively, and the contribution to clinical judgment may therefore be limited. The combination of single markers overcame this problem and clearly stratified patients into low‐, intermediate‐, and high‐risk groups 10, 32, 33. Low‐risk patients expected to achieve excellent long‐term outcomes will therefore avoid excessive intervention associated with monitoring and treatment that can reduce a patient's burden and medical costs. In contrast, identification of patients at high risk of recurrence with an adverse prognosis is helpful to physicians for making management decisions, allowing selection of patients eligible for intensive follow‐up and treatment.

*SYT8* contributes to the trafficking and exocytosis of secretory vesicles in non‐neuronal tissues, and *SYT8* expression in human pancreatic islets is associated with the activity of the promoter of the insulin gene 34, 35. Further, *SYT8* is a candidate biomarker specific for peritoneal metastasis, according to the results of a recurrence pattern‐specific transcriptome analysis of patients with stage III GC who underwent curative gastrectomy and adjuvant S‐1 monotherapy 24. *MAGED2* plays a role in cell adhesion, and increased expression of *MAGED2* is associated with nodal and hematogenous metastasis and is an independent prognostic factor for gastric cancer 17, 36. The distinct roles of *SYT8* and *MAGED2* in the progression of gastric cancer synergistically enhanced predictive performance, achieving stratification that is more precise. With respect to recurrences after curative gastrectomy, patients at high risk of peritoneal and nodal recurrences were identified by the dual‐marker expression panel possibly because the panel could synergistically enhance the linkages of the two constituent biomarkers to differential malignant phenotypes of gastric cancer. Moreover, our expression score is advantageous, because it can be determined using only two markers and is therefore more convenient and cost‐effective compared with existing diagnostic techniques.

In the present study, resected gastric tissues were used to measure the expression levels of molecular markers. As endoscopic biopsy samples are also available for mRNA analysis and immunohistochemistry, expression scores can be determined before surgery and may contribute to decision‐making regarding the indication of neoadjuvant treatment or staging laparoscopy as well as the selection of a surgical procedure. Although mRNA expression levels were used because they are easy to quantitate, immunohistochemical detection *in situ* of SYT8 and MAGED2 was achieved in our previous studies 17, 24. Moreover, the significant correlations between staining intensity and qPCR results were demonstrated both in studies for *SYT8* and *MAGED2* 17, 24. The use of readily available and commonly used clinical immunohistochemical techniques should be considered 37. Moreover, immunohistochemistry data might merit inclusion as a criterion for prospective clinical trials that evaluate the survival benefit of neoadjuvant treatment or adjuvant combination chemotherapy. Finally, in the current era of patient‐centered communication and shared decision‐making, providers are expected to actively engage patients more frequently in decisions, using their own medical knowledge and quantitative expression data.

The limitations of the present study include its retrospective design, relatively small sample size, and the long period of study at 13 years. qRT‐PCR results were normalized using only GAPDH as a housekeeping gene, although it was reported that GAPDH might be influenced by oxidant conditions 38. Despite an effort to reduce selection bias using a two‐step evaluation, additional validation of the utility of the dual‐marker expression panel by future large‐scale prospective studies is required for optimization of cutoff values and widespread translation to clinical practice. Nevertheless, this study concept can leverage current knowledge of single molecular markers and bring it to the next stage, which represents an important step forward in the realization of precision surgery.

In summary, the dual‐marker expression panel comprising two original molecular markers is simple and cost‐effective for risk stratification of patients with gastric cancer. We expect that this concept will maximize the predictive performance of single markers to improve risk stratification and enhance personalized surgical oncology.

## Conflict of Interest

None declared.

## Supporting information


**Figure S1.** The prognostic value of the preoperative serum (A) CEA and (B) CA19‐9 levels in the validation set.

**Table S1**. Primers used for quantitative RT‐PCR.

**Table S2.** Characteristics of patients in the learning and validation sets.

**Table S3.** Evaluation of the dual‐marker expression panel to predict overall survival.

**Table S4.** Prognostic factors for disease‐free survival of patients who underwent curative resection in the validation set.
