# Supplementary figures and images for: A novel dual‐marker expression panel for easy and accurate risk stratification of patients with gastric cancer
Source: Cancer Med. 2018 May 7;7(6):2463–71. doi: 10.1002/cam4.1522 (PMC6010733; doi:10.1002/cam4.1522)

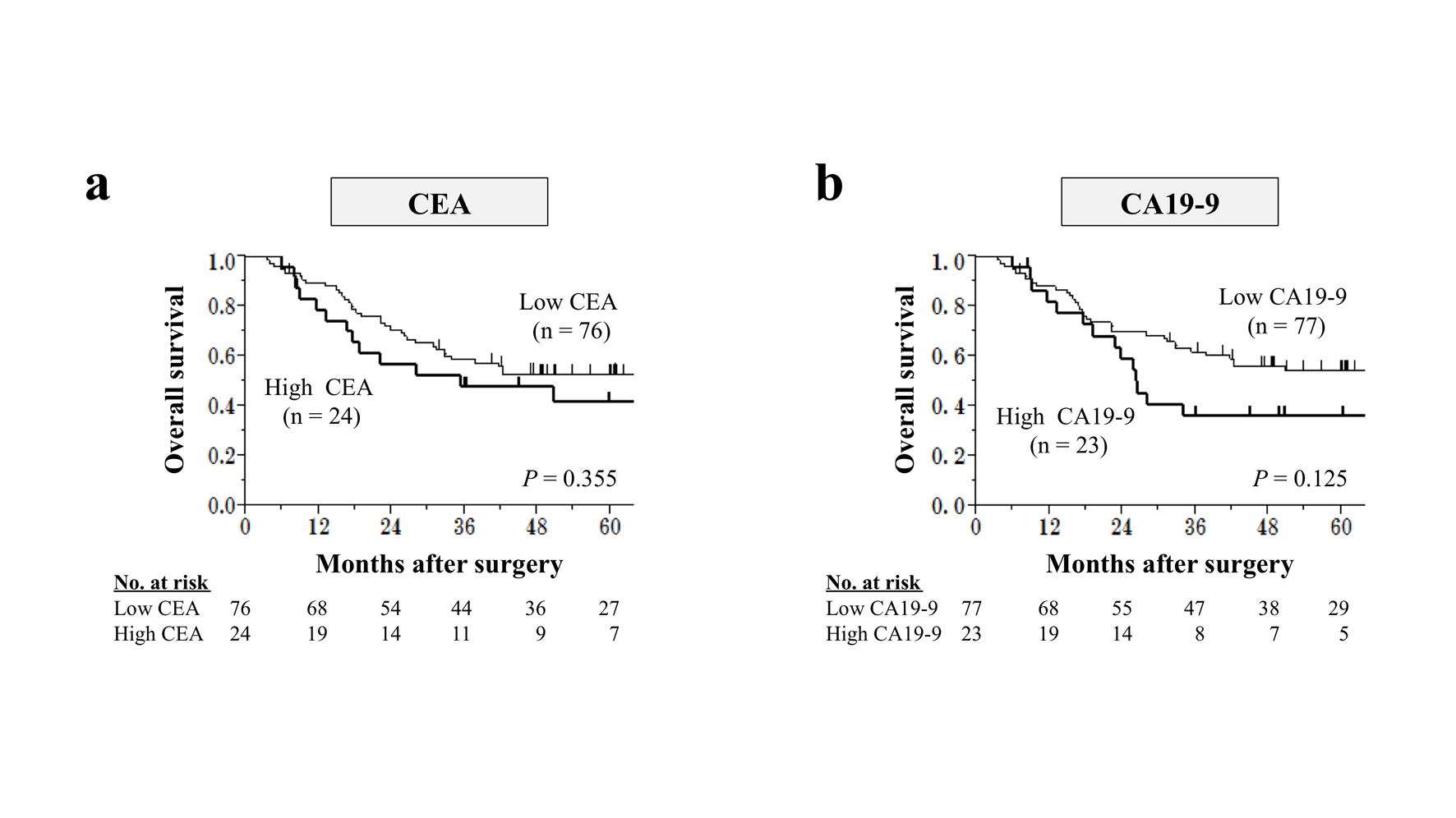

Supplement: Supplementary file 1 — Figure S1. The prognostic value of the preoperative serum (A) CEA and (B) CA19‐9 levels in the validation set. [file CAM4-7-2463-s001.tif]
