# Supplementary material for: A novel dual‐marker expression panel for easy and accurate risk stratification of patients with gastric cancer
Source: Cancer Med. 2018 May 7;7(6):2463–71. doi: 10.1002/cam4.1522 (PMC6010733; doi:10.1002/cam4.1522)
Supplement: Supplementary file 2 — Table S1. Primers used for quantitative RT‐PCR. [file CAM4-7-2463-s002.docx]

**Supplementary Table 1.** Primers used for quantitative RT-PCR

|  | **Type** | **Nucleotide sequences (5´ - 3´)** | **Size** |
| --- | --- | --- | --- |
| *ANOS1* | forward | AACAATGGTTCCCTGGTTTG | 110 bp |
|  | reverse | TCACAAAAGCTTTGGCACTG |  |
| *DPYSL3* | forward | AGAAGAAGGAGGGAGGGAGC | 110 bp |
|  | reverse | CTCCCTTGATAAGGAGACGG |  |
| *BTG1* | forward | CTGCAGACCTTCAGCCAGA | 104 bp |
|  | reverse | CGAATACAACGGTAACCCGA |  |
| *MZB1* | forward | CTCACAGGCCCAGGACTTAG | 219 bp |
|  | reverse | TGTGGCTGACACCTTCTCTG |  |
| *SAMSN1* | forward | TGCTCAAGAGAAAGCCATCC | 97 bp |
|  | reverse | TTATTCCGAAAACGATCGAAA |  |
| *DENND2D* | forward | CACTGCTCTACCCCTTCAGC | 204 bp |
|  | reverse | TTTTTCATCACCAACCGACA |  |
| *GPR155* | forward | AGCAAAGCTGGACTATTCCCT | 125 bp |
|  | reverse | GCCACCAAATAAATGTACTGGA |  |
| *MFSD4* | forward | CAACATGCAGCTGGTAAGGA | 192 bp |
|  | reverse | ACCCTGGAGACATGGAACAG |  |
| *SYT8* | forward | GCTTCTCTCTCCGGTACGTG | 196 bp |
|  | reverse | AGGAAGGTGAAGGCCTCATT |  |
| *PDSS2* | forward | GAATCAGGTAGTGTCAGAGG | 181 bp |
|  | reverse | GAGGCTATTCCAGCTGTCATG |  |
| *FAM46C* | forward | CATGTGGCTCTTCCAACAGA | 219 bp |
|  | reverse | CTTCAGCTCCACGTTCTTCC |  |
| *PRMT5* | forward | TCTCATGGTTTCCCATCCTC | 102 bp |
|  | reverse | CCTTCTTGGAATTGCTGCAT |  |
| *NRAGE* | forward | GATTCCCTCAGACCTTTGC | 170 bp |
|  | reverse | GAAGGAATCTGAGGCTTCAG |  |
| *MAGED2* | forward | TAGAGAAGGCAGACGCATCC | 110 bp |
|  | reverse | AAGCGAGTTAGACCTGCACC |  |
| *TUSC1* | forward | ACATGTACAGTTCCCCTGCC | 110 bp |
|  | reverse | GCCACCAAATAAATGTACTGGA |  |
| *GAPDH* | forward | GAAGGTGAAGGTCGGAGTC | 226 bp |
|  | probe | CAAGCTTCCCGTTCTCAGCC |  |
|  | reverse | GAAGATGGTGATGGGATTTC |  |
