# Supplementary material for: A novel dual‐marker expression panel for easy and accurate risk stratification of patients with gastric cancer
Source: Cancer Med. 2018 May 7;7(6):2463–71. doi: 10.1002/cam4.1522 (PMC6010733; doi:10.1002/cam4.1522)
Supplement: Supplementary file 3 — Table S2. Characteristics of patients in the learning and validation sets. [file CAM4-7-2463-s003.docx]

**Supplementary Table 2.** Characteristics of patients in the learning and validation sets

|  | **Learning set**  **(n=100)** | **Validation set**  **(n=100)** | ***P*** |
| --- | --- | --- | --- |
| Age (years), mean ± SD | 65.6 ± 11.5 | 65.7 ± 11.0 | 0.955 |
| Sex (male/female) | 70 / 30 | 71 / 29 | 0.877 |
| Tumor location  Entire  Upper third  Middle third  Lower third | 9  23  32  36 | 9  19  27  45 | 0.613 |
| Macroscopic type  Borrmann type 4/5  Others | 20  80 | 15  85 | 0.351 |
| UICC stage  I  II  III  IV | 18  18  28  36 | 16  15  34  35 | 0.805 |
| Follow up months, median | 61.9 | 60.9 | 0.617 |

*Abbreviations*: *SD*, standard deviation.
