# Supplementary material for: A novel dual‐marker expression panel for easy and accurate risk stratification of patients with gastric cancer
Source: Cancer Med. 2018 May 7;7(6):2463–71. doi: 10.1002/cam4.1522 (PMC6010733; doi:10.1002/cam4.1522)
Supplement: Supplementary file 4 — Table S3. Evaluation of the dual‐marker expression panel to predict overall survival. [file CAM4-7-2463-s004.docx]

**Supplementary Table 3.** Evaluation of the dual-marker expression panel to predict overall survival

| **C-index;**  **dual-marker panel** | **Marker** | **C-index;**  **single marker** | **Coefficient** | **HR** | **95% CI** | ***P*** |
| --- | --- | --- | --- | --- | --- | --- |
| 0.718 | *SYT8* | 0.653 | 1.222 | 3.394 | 1.703-6.766 | 0.001 |
|  | *MAGED2* | 0.612 | 0.902 | 2.466 | 1.195-5.086 | 0.015 |

*Abbreviations*: *HR*, hazard ratio; *CI,* confidence interval.
