# Supplementary material for: A novel dual‐marker expression panel for easy and accurate risk stratification of patients with gastric cancer
Source: Cancer Med. 2018 May 7;7(6):2463–71. doi: 10.1002/cam4.1522 (PMC6010733; doi:10.1002/cam4.1522)
Supplement: Supplementary file 5 — Table S4. Prognostic factors for disease‐free survival of patients who underwent curative resection in the validation set. [file CAM4-7-2463-s005.doc]

**Table S4 Prognostic factors for disease-free survival of patients who underwent curative resection in the validation set**

| **Variables** | **Univariate** | | | **Multivariate** | | |
| --- | --- | --- | --- | --- | --- | --- |
|  | **Hazard ratio** | **95% CI** | ***P*** | **Hazard ratio** | **95% CI** | ***P*** |
| Age (≥ 70) | 0.80 | 0.32–1.87 | 0.616 |  |  |  |
| Gender (male) | 1.64 | 0.65–4.98 | 0.312 |  |  |  |
| CEA (> 5 ng/ml) | 1.24 | 0.36–3.32 | 0.708 |  |  |  |
| CA19-9 (> 37 IU/ml) | 2.76 | 0.99–6.75 | 0.053 |  |  |  |
| Tumor location (lower third) | 0.99 | 0.43–2.35 | 0.989 |  |  |  |
| Tumor size (≥ 50 mm) | 1.77 | 0.75–4.64 | 0.199 |  |  |  |
| Tumor depth (pT4, UICC) | 2.02 | 0.87–4.78 | 0.102 |  |  |  |
| Tumor differentiation (undifferentiated) | 1.17 | 0.50–2.93 | 0.720 |  |  |  |
| Lymphatic involvement | 4.69 | 0.98–84.0 | 0.054 |  |  |  |
| Vascular invasion | 3.80 | 1.50–11.6 | 0.004 | 2.72 | 1.04–8.54 | 0.041 |
| Invasive growth | 1.75 | 0.72–4.07 | 0.206 |  |  |  |
| Lymph node metastasis | 3.79 | 1.41–13.1 | 0.007 | 1.23 | 0.38–4.99 | 0.751 |
| UICC stage (III) | 4.11 | 1.62–12.5 | 0.002 | 2.83 | 0.97–9.99 | 0.057 |
| Expression score (1 or 2) | 5.23 | 1.78–22.3 | 0.002 | 4.24 | 1.42–18.3 | 0.008 |

CEA, carcinoembryonic antigen; CA19-9, carbohydrate antigen 19-9; UICC, Union for International Cancer Control.
